# Obituary

**Published:** 1883-04

**Authors:** 


### OBITUARY.

Died at Belfast, N. Y., February 24, 1883, John H. Saunders, M. D., aged 63 years.

Dr. Saunders was a delegate at the last meeting of the State Medical Society, and from undue exertion and exposure fell a victim to typhoid pneumonia. He was held in high esteem in the section in which he practiced, and was widely known as a highly educated and skillful physician. The profession, of which he was an earnest member, and the community to which he devoted the best energies of mind and body, feel the loss they have sustained in the death of this estimable gentleman and conscientious practitioner.

